# COVID-19: Current Developments and Further Opportunities in Drug Delivery and Therapeutics

**DOI:** 10.3390/pharmaceutics12100945

**Published:** 2020-10-03

**Authors:** Saman Zafar, Muhammad Sohail Arshad, Sameen Fatima, Amna Ali, Aliyah Zaman, Elshaimaa Sayed, Ming-Wei Chang, Zeeshan Ahmad

**Affiliations:** 1 Faculty of Pharmacy, Bahauddin Zakariya University, 60800 Multan, Pakistan; samanzafarawan@gmail.com (S.Z.); sohail_arshad79@yahoo.com (M.S.A.); sameenf805@gmail.com (S.F.); 2 Leicester School of Pharmacy, De Montfort University, Leicester LE1 9BH, UK; p15182582@alumni365.dmu.ac.uk (A.A.); aliyah-454@hotmail.co.uk (A.Z.); alshimaa_gamal_2008@hotmail.com (E.S.); 3 Nanotechnology and Integrated Bioengineering Centre, University of Ulster, Northern Ireland BT37 0QB, UK; m.chang@ulster.ac.uk

**Keywords:** SARS-CoV-2, COVID-19, coronavirus, infection, immunization, immune response, vaccine, drug delivery

## Abstract

SARS-CoV-2 has affected people from all age groups, races and ethnicities. Given that many infected individuals are asymptomatic, they transmit the disease to others unknowingly, which has resulted in the spread of infection at an alarming rate. This review aims to provide an overview of the pathophysiology, preventive measures to reduce the disease spread, therapies currently in use, an update on vaccine development and opportunities for vaccine delivery. The World Health Organization has advised several precautions including social distancing, hand washing and the use of PPE including gloves and face masks for minimizing the spread of SARS-CoV-2 infection. At present, several antiviral therapies previously approved for other infections are being repositioned to study their efficacy against SARS-CoV-2. In addition, some medicines (i.e., remdesivir, chloroquine, hydroxychloroquine) have received emergency use authorisation from the FDA. Plasma therapy has also been authorised for emergency use for the treatment of COVID-19 on a smaller scale. However, no vaccine has been approved so far against this virus. Nevertheless, several potential vaccine targets have been reported, and development of different types of vaccines including DNA, mRNA, viral vector, inactivated, subunit and vaccine-like particles is in process. It is concluded that a suitable candidate delivered through an advanced drug delivery approach would effectively boost the immune system against this coronavirus.

## 1. Introduction to Severe Acute Respiratory Syndrome Coronavirus 2 (SARS-CoV-2) Infection

In the 21st century, corona viruses (CoVs) have caused three major outbreaks including severe acute respiratory syndrome, SARS-CoV-1 or SARS-CoV (2002) [1]; middle east respiratory syndrome, MERS CoV (2012) [2]; and SARS-CoV-2 or coronavirus disease, COVID-19 (2019) [3]. Amongst these, COVID-19 was considered as a worldwide pandemic. CoVs are generally categorized into two major groups including alpha-CoVs (HCoV-229E and HCoV-NL63) and beta-CoVs (HCoV-OC43 and HCoV-HKU1). Reports suggest that CoVs responsible for the three outbreaks mentioned above are beta-CoVs [4]. The current pandemic, COVID-19, emerged as an outbreak of pneumonia, initially with an unknown aetiology, in December 2019, from a seafood market in Wuhan (Hubei province, China). The genome of SARS-CoV-2, the virus responsible for COVID-19, showed >80% resemblance with SARS-CoV [5,6,7]. As per World Health Organization (WHO) statistics, up until the 28th of September 2020, coronavirus infection has spread into 235 territories with ~33 million infected individuals and ~1 million deaths (~3%) [8]. This novel virus is less deadly than SARS-CoV-1 (9.7%) and MERS CoV (34%), but relatively highly transmissible and hence termed as a pandemic [9].

## 2. Transmission

The emergence of SARS-CoV-2 from a wet animal market suggests its zoonotic origin [10,11]. Researchers are trying to determine a possible reservoir host/intermediate carrier that may have passed the infection onto humans. Earlier reports suggested two species of snakes as possible hosts of the virus [12]. Genome sequence analysis suggests its 88 and 50% similarity to bat-derived SARS and MERS CoVs, respectively, suggesting mammals as the most probable link between the virus and humans. Many reports confirmed person to person transmission as a major cause of its spread. This transmission can take place by direct contact or through respiratory droplets (coughing and sneezing) of an infected individual [12,13]. Earlier, in March 2020, it was reported that COVID-19 is not transmitted through air, after an analysis of >75,000 COVID-19 patients in China. However, airborne transmission may take place during aerosol producing procedures such as endotracheal intubation, bronchoscopy, administration of nebulized treatment, turning the patient to the prone position, open suctioning and cardiopulmonary resuscitation. Later on, in July 2020, a few outbreaks were reported in indoor crowded spaces, suggesting the chances of aerosol transmission in combination with droplet transmission [14,15]. Reports suggest that this virus transmits due to linking of receptor-binding domain (RBD) of a virus spike with a cellular receptor angiotensin converting enzyme 2 (ACE2). The RBD of the SARS-CoV-2 spike resembles the SARS-CoV spike [12,13,16].

Pregnant women are relatively more prone to respiratory infections [12]. Recently, Vivanti et al. reported transplacental transfer of current SARS-CoV-2 in a neonate born to an infected mother. The neonate presented with neurological indications, similar to those exhibited by adult patients [17].

## 3. Pathogenesis

Lin et al. proposed a hypothetical pathogenesis of COVID-19 infection in humans on the basis of published literature, as well as clinical observations of infected individuals, and divided the disease into three clinical phases. The virus could possibly move through the mucosal membranes (nasal/larynx mucosa) and invade the lungs via the respiratory tract, where it then passes into the peripheral blood. After entering the blood, the virus may attack ACE2-expressing organs including lungs, gastrointestinal tract, kidneys and the heart. The authors suggested that detection of SARS-CoV-2 in faecal samples supports their hypothesis, as presence of the virus in faeces is most likely due to the movement of the virus from the lungs into the blood and finally the intestines [18].

**Figure 1:**
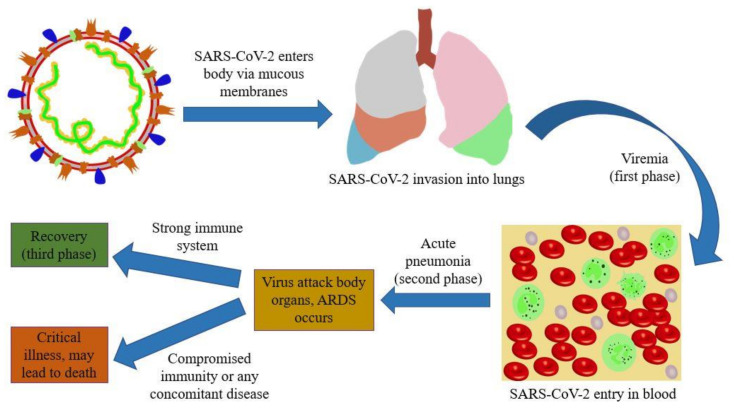
Clinical phases of SARS-CoV-2 infection.

The average time period, from onset of symptoms to acute respiratory distress syndrome (ARDS), is ~8 days. If the patient’s immune system is effective in the second phase and there is no concomitant disease, the virus can be suppressed efficiently, and the individual can recover (Figure 1). If an infected individual is older, in an immunocompromised condition or experiencing any concomitant condition, then the virus might attack again, leading to aggravated symptoms within 7–14 days after onset [18]. Various potential risk factors for the severity of infection may include obesity, host genetics, pregnancy, smoking and many more. However, there is still limited data available about other factors that might be reasons for the variation of the severity of the disease [19,20]. Reports suggested host innate immune response as higher neutrophil, C-reactive protein, interleukin-6 (IL-6) and reduced lymphocyte counts were observed in infected individuals. Neutrophilia and lymphocytopenia resulted in increased disease severity [18,21,22].

Type I interferon (IFN-α, IFN-β) promotes suppression of viral replication/dissemination, but in the case of SARS-CoV-2 infection, responses from these interferons were found to be suppressed. The humoral immune response plays a key role in suppressing infection at a subsequent stage and prevents re-infection by producing antibodies. IgG antibodies against N protein were detected in SARS-CoV-2-infected individuals after ~4 days of onset of the disease [23]. Significantly low B lymphocyte count possibly affected antibody production in critically ill patients. T-cell immune response against SARS-CoV-2 infection is required to recognize and kill infected cells, especially in the lungs. CD8+ T lymphocytes combat infected cells while T helper cells produce many inflammatory cytokines [12,23,24]. COVID-19 patients exhibited both CD4+ and CD8+ T cell responses. In fact, multiple distinct response patterns may be detected in different individuals, suggesting the likelihood of particular clinical approaches tailored to the specific immunotype of an individual. Recently, a preprint study reported that T cell mediated immunity was observed in SARS-CoV-2-infected individuals (with no or mild symptoms), even when there was a negative antibody test. This suggests that cellular immunity may play a more significant role against COVID-19 compared to antibody-mediated immunity. Early data regarding memory T cell responses are promising as CD4+ and CD8+ T memory cells were found in 100% and 70% of the recovered individuals, respectively. Furthermore, memory T cell responses were also observed against many SARS-CoV-2 proteins, i.e., spike protein, nucleoprotein and membrane protein [25,26,27,28]. Infected individuals exhibited significant increases in infection related pro-inflammatory cytokines (IL-7, IL-6, IL-2, IL-10, IP-10, TNFα, GCSF, MCP1 and MIP1α), which possibly contributed to the severity of symptoms within 7–14 days after onset. Severe patients exhibited elevated creatinine, erythrocyte sedimentation rate, D-dimer and blood urea nitrogen levels [29,30].

Recently, a case of re-infection was reported by To et al. in an apparently immunocompetent individual who developed asymptomatic re-infection, by a different SARS-CoV-2 strain, ~4.5 months after the first symptomatic infection. This suggested that this virus may continue to transmit in humans despite development of herd immunity via natural infection and/or vaccination [31].

## 4. Diagnosis and Symptoms

Reverse transcriptase polymerase chain reaction (RT-PCR) is the test performed to identify positive nucleic acid of the virus in samples obtained from throat swabs, sputum and lower respiratory tract secretions [32]. Many individuals infected with SARS-CoV-2 do not experience any symptoms (asymptomatic). As per the World Health Organization (WHO), 27 May 2020, most infected individuals develop mild (40%), moderate (40%) or severe symptoms (~15%), while only 5% suffer from critical disease (Table 1) [33,34]. Published data showed an average incubation period for appearance of symptoms as ~5.2 days. In a study, time duration from onset of symptoms to death was ~6–41 days (median 14 days), but this duration was influenced by the patient’s age (shorter in patients > 70 years of age) and immune response [12,35].

Infected individuals showed respiratory symptoms including rhinorrhoea, sore throat, sneezing, pneumonia, acute respiratory distress syndrome (ARDS), RNA-aemia and ground-glass opacities in the sub-pleural region of the lungs. These opacities possibly induced immune responses (systemic as well as localized), resulting in elevated inflammation, eventually leading to death. Various systemic symptoms such as fever, cough, sputum production, diarrhoea, lymphopenia, dyspnoea, haemoptysis, fatigue, headache and acute cardiac injury were also seen in patients [12,35,36,37].

## 5. Prevention

WHO recommends several precautions to reduce chances of getting infected or spreading SARS-CoV-2. These precautionary measures include regular and thorough hand washing using soap or alcohol-based sanitizer, maintaining at least one-meter distance from others, using a face mask, avoiding touching the eyes, nose or mouth and going to crowded areas [38,39]. For suspected individuals, experiencing mild symptoms such as fever <38 °C (goes down by itself), cough, no asthma, dyspnoea or an underlying chronic condition, in-house isolation and care has been recommended [40].

According to the University of Oxford’s COVID-19 Evidence Service Team, SARS-CoV-2 has an R_0_ (basic reproduction number) of ~2.63 (range 0.4–4.6) [41]. In addition, it has an Re (essential reproductive number) of >1, which shows that the outbreak is expected to continue. Current SARS-CoV-2 infection statistics, especially in China, suggests that the advised precautionary measures can cause a decline in R-values [41,42]. Once the number of cases become manageable, more precise measures including contact tracing or location tracing can be used to restrict the R-values [43]. In a few countries, after lockdown easing measures, the Re rose again to be more than 1 [41]. Earlier, it was speculated that the R-value would significantly decrease in warm weather, but as per WHO, SARS-CoV-2 infection can spread regardless of the weather; thus, the best way to reduce infection rate is to practice social distancing and other safety measures [44].

## 6. Pharmacologic Treatments

Treatment of co-existing indications such as hypertension, asthma, diabetes, chronic obstructive pulmonary disease, renal disease or liver disease should be continued [45]. Proper mechanical ventilation should be provided to patients experiencing breathing difficulties [46]. Many therapies currently administered or prescribed to SARS-CoV-2-infected individuals include antiviral, plasma, anti-inflammatory, antimicrobial and anticoagulant therapies.

### 6.1. Antiviral Therapy

As treatment of COVID-19 is urgently required, one possible strategy is to repurpose existing anti-viral agents against the current COVID-19 infection [47,48]. Sayad et al. suggested the use of sofosbuvir, an approved anti-hepatitis virus C agent, as a repurposed antiviral drug against SARS-CoV-2 infection [49]. Recently, nitazoxanide, a broad-spectrum antiviral and antiparasitic agent, emerged as a potential repositioned drug for treating COVID-19 patients [50]. Various antiviral agents including lopinavir/ritonavir, remdesivir, ribavirin, favipiravir, arbidol, IFN-α and chloroquine/hydroxychloroquine have been repurposed specifically for the management of COVID-19 (Table 2).

In genomic analysis, SARS-CoV-2 expressed four enzymes, including RNA-dependent RNA polymerase (RdRp), papain-like protease (PLpro), chymotrypsin-like protease (3CLpro) and helicase, exhibiting thoroughly conserved catalytic sites/drug binding pockets and resemblance with SARS/MERS CoVs sequences. Homology modelling revealed that a SARS-CoV-2 protease, known as endopeptidase C30 (CEP_C30), binds efficiently with protease inhibitors, i.e., lopinavir and ritonavir, suggesting anti-SARS-CoV-2 activity of drugs probably due to their inhibitory action on C30 (CEP_C30). Lopinavir/ritonavir showed antiviral activity against SARS-CoVs in cell culture, but results against MERS CoVs were conflicting [51,52]. Cao et al. revealed that the condition of SARS-CoV-2-infected individuals did not improve by using these protease inhibitors [53].

Remdesivir is a nucleotide antiviral prodrug and was previously used to treat Ebola virus infections [54]. In August 2020, the Food and Drug Administration (FDA) broadened emergency use authorisation for remdesivir and recommended its use for all COVID-19 hospitalized patients, irrespective of infection severity [55]. Sheahan et al. reported an improvement in COVID-19 treatment outcomes and reduction in SARS-CoV-2 viral loads in a mouse model using remdesivir [56]. Ribavirin is another antiviral agent that has been used for the treatment of hepatitis C infections and some viral haemorrhagic fevers. Ribavirin showed in-vitro antiviral activity against SARS-CoV-2 infection, but a major limitation associated with this drug is the reduction of haemoglobin levels, which is undesirable in patients suffering respiratory distress [57]. Sheahan et al. compared the activity of remdesivir, IFNb, lopinavir and ritonavir in-vitro and in-vivo against MERS CoV. The results showed that remdesivir and IFNb exhibited higher antiviral activity than lopinavir and ritonavir in-vitro. In the mouse model, remdesivir showed significant improvement in pulmonary function and reduced lung viral loads compared to lopinavir/ritonavir/IFNb combination therapy [58]. Hung et al. reported a trial carried out in SARS-CoV-2-infected human subjects in Hong Kong. Patients in the combination group were delivered interferon beta-1b/ribavirin/lopinavir and ritonavir combination therapy, while control group patients were administered ritonavir and lopinavir. The combination group exhibited a shorter median time (7 days) from the beginning of the treatment to negative nasopharyngeal swab as compared to the control group (12 days) [59].

Nucleoside analogues of the guanine derivative, favipiravir, approved in Japan (2014) for the treatment of pandemic influenza infection, can be efficiently used to inhibit RdRp of influenza virus, flavivirus, enterovirus, norovirus and Ebola virus, in turn, blocking viral RNA synthesis [51,60]. Its antiviral activity against SARS-CoV-2 was modest in the cell culture. Results of an open labelled, non-randomized study (*n* = 35–45) showed viral clearance time in favipiravir treated patients as 4 days, which was sufficiently lower than the control group (11 days) treated using a combination therapy comprised of lopinavir/ritonavir and IFN-α [61,62]. Arbidol, an approved antiviral candidate in Russia and China, displays activity against influenza virus. A study conducted on SARS-CoV-2-infected individuals with mild symptoms treated using arbidol and favipiravir suggested cough relief, fever reduction and a higher clinical recovery rate in the favipiravir treated group; however, results were not promising in critically ill patients [62,63].

Antimalarial drugs, i.e., chloroquine and hydroxychloroquine, were prescribed to infected individuals, but a study published in *The Lancet* reported increased fatalities and heart issues in some infected individuals. WHO immediately halted trials of these drugs but resumed trials when *The Lancet* withdrew the study due to shortcomings. The FDA allowed emergency use of chloroquine/hydroxychloroquine in March 2020 but withdrew the drugs later on in June [64,65,66]. In an update published by Cochrane, in June 2020, low certainty evidence indicated that hydroxychloroquine may cause more serious adverse events than standard treatment for COVID-19 [67]. Moreover, there were reports of individuals poisoning themselves by using these drugs without proper medical supervision. WHO cautioned people to avoid self-medication as well as physicians prescribing drugs that had not been proven to treat SARS-CoV-2 [64,65,68].

**Table 2:** Antiviral agents, repurposed for SARS-CoV-2 infection.

Antiviral Agent	Mechanism of Action	Activity Against SARS-CoV-2	FDA Pregnancy Category	Usage in Renal Dysfunction	Usage in Hepatic Dysfunction	References
Lopinavir/Ritonavir	Inhibits 3CLpro protease activity of virus	Reduced viral loads	C (lopinavir), B (ritonavir)	+	++	[69,70,71]
Remdesivir	Inhibitor of DNA/RNA polymerases, competes with ATP substrate for inclusion into nascent RNA chains resulting into delayed chain termination during viral RNA replication	Inhibited SARS-CoV-2 in-vitro, shortened the time to recovery	**	+++	+++	[71]
Ribavirin	Interferes with DNA/RNA replication, interferes with RNA capping, inhibits natural guanosine production	Showed activity against SARS-CoV-2 in-vitro	X	##	+	[72,73,74,75,76]
IFN-α	Inhibits viral synthesis, elicits natural immune responses	Used in combination with lopinavir/ritonavir and favipiravir against virus	C	+	Not recommended in patients suffering from autoimmune hepatitis, decompensated liver disease	[77,78]
Favipiravir	Perceived as a purine nucleotide, mistakenly, by the viral RNA polymerase	Inhibited virus in-vitro, rapid viral clearance and better treatment effects on infected individuals	**	+	## (in severe impairment)	[71]
Arbidol	Impedes virus-mediated fusion, inhibits viral invasion into the target cells, targets S-protein of SARS-CoV-2 and impedes its trimerization	Reduced mortality, its post-exposure prophylaxis served as a protective factor against COVID-19 development, showed antiviral effects against virus in combination with lopinavir/ritonavir	**	++ (in severe impairment)	++	[71]
Chloroquine phosphate	Inhibits viral infection by raising endosomal pH needed for cell fusion, interferes with the glycosylation of cellular receptors	Showed activity against SARS-CoV-2 in-vitro	**	##	++	[71,79]
Hydroxy- chloroquine	Possibly resembles chloroquine	Exhibited superior potency compared to chloroquine in-vitro, reduced the time to recovery, promoted the absorption of pneumonia	**	##	++	[71,80]

+ usable, ++ use with caution, +++ use after accessing risk benefit ratio, ** to the best of authors knowledge, no category assigned, formally, by FDA, ## dose reduction/adjustment required.

### 6.2. Plasma Therapy

Plasma therapy, a passive immunotherapy method, has previously been used for SARS/MERS pandemics. FDA issued emergency use authorisation for this therapy on 23 August, 2020 [81]. A possible explanation for the effectiveness of this treatment approach is that the antibodies present in the plasma of recovered individuals might suppress viremia [57]. Guo et al. found high concentrations of SARS-CoV antibodies that could remain for ~12 years in cured individuals and suggested that similar antibodies would possibly have some therapeutic efficacy against SARS-CoV-2 infection [82]. Currently, many people have been cured from SARS-CoV-2 infection; therefore, plasma therapy can serve as a safe therapeutic approach as long as sufficient antibody titre is sustained. Clinical trials reported sufficient effectiveness of plasma therapy for SARS-CoV-2-infected individuals. Treatment of critically ill individuals using plasma therapy resulted in amelioration of their clinical status [51,83]. However, there is a risk of aggravating hyperimmune responses and transmission of serious infections such as hepatitis, syphilis and HIV. The transfusing antibodies provide immunity for a short duration (few weeks). Plasma therapy is relatively more effective in the earlier stages of illness (up to ~14th day) [84,85]. Moreover, plasma therapy is subjected to sourcing issues and ethical restrictions. Currently, it is difficult to promote its widespread use due to inadequacy of collecting large samples, validation of the individual samples and lack of evidence from properly designed clinical trials [51,83].

### 6.3. Anti-Inflammatory Therapy

Use of anti-inflammation therapy in COVID-19 patients may help in treating the cytokine storm, which leads to ARDS. Numerous anti-inflammatory agents such as NSAIDS (non-steroidal anti-inflammatory drugs), glucocorticoids and inflammatory cytokine antagonists (e.g., janus kinase (JAK) inhibitors) have been considered for anti-inflammation therapy in COVID-19. A critical issue regarding anti-inflammatory therapy is balancing the risk/benefit ratio [86].

Efficacy of NSAIDs is still unknown; there have been warnings and consequent confusion regarding their use in COVID-19 patients due to their adverse effects. As per a WHO briefing (13 April 2020), no conclusive evidence of serious side effects due to the use of NSAIDS in SARS-CoV-1-, MERS CoV- and SARS-CoV-2-infected individuals exists, but this lack of evidence does not assure absence of severe adverse effects [87]. On 16 March 2020, French authorities recommended the use of acetaminophen (paracetamol) instead of ibuprofen on the basis of evaluation of four COVID-19 patients [88,89].

Adverse effects associated with corticosteroid therapy, such as delayed viral clearance and secondary infection risk, restricts their use in SARS-CoV-2-infected individuals [90]. However, on 16 June 2020, it was revealed by the investigators of COVID-19 recovery trials that dexamethasone administration to SARS-CoV-2-infected individuals with severe symptoms resulted in 8–26% lower mortality as compared to the individuals given the usual therapy. Dexamethasone therapy increased 28-day survival among the hospitalized patients receiving invasive mechanical ventilation or oxygen therapy. Trial results suggested that despite concerns regarding steroid-associated side effects, dexamethasone can serve as a potential therapy (readily available with low cost) against SARS-CoV-2 infection [91].

JAK inhibitors block INFα production, which is significant in fighting virus, and thus may not be appropriate for treating the inflammatory cytokine storm. Baricitinib, a JAK inhibitor, was suggested for COVID-19 patients. Currently, clinical trials of two JAK inhibitors including jakotinib hydrochloride and ruxolitinib have been registered for treating COVID-19 patients [86].

### 6.4. Antimicrobial Therapy

Antimicrobial agents are also being administered to SARS-CoV-2-infected individuals to combat bacterial (*Mycoplasma pneumoniae*), fungal (Candida species) and viral (HIV/influenza/rhinovirus) co-infections. Careful administration of broad-spectrum antibacterial agents (macrolides, fluoroquinolones, tetracyclines, β-lactam antibiotics, etc.) against commonly observed infections (caused by *Klebsiella pneumoniae*, *Staphylococcus aureus* (methicillin-resistant), *Streptococcus pneumoniae* (multi-drug resistant), *Pseudomonas aeruginosa* and *Acinetobacter baumannii* species) in infected individuals, undergoing hospitalization (>6 days), is recommended [57,92].

### 6.5. Anticoagulant Therapy

Many studies reported SARS-CoV-2 infection-associated coagulopathy which have been detected by elevated D-dimer, fibrin/fibrinogen degradation products. However, deviations in the platelet count, activated partial thromboplastin and prothrombin time were less common. Administration of anticoagulant agents such as unfractioned or low molecular weight heparins (e.g., enoxaparin) for prophylaxis or management of thromboembolism, disseminated intravascular coagulopathy or sepsis-induced coagulopathy is recommended. Bleeding is still not reported in SARS-CoV-2-infected patients. If any such cases occur, then standard protocols for the management of coagulopathy and bleeding should be followed. Heparin therapy should also be continuously monitored by measuring activated partial thromboplastin time/anti-factor Xa levels, as its overdose can result in bleeding [93,94,95,96].

## 7. Immunization

**Figure 2:**
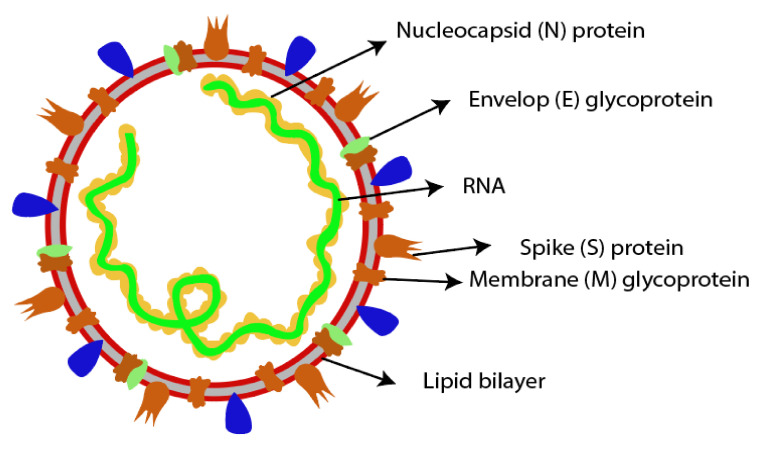
Representative structure of human coronavirus.

Currently, the preferred therapy for infectious diseases includes the use of antibiotics; however, failure of antibiotic therapy due to resistance has resulted in the re-emergence of various infections. Rapid migration of humans, animals and raw materials around the globe is accelerating the spread of infections and leading to pandemics, e.g., the recent SARS-CoV-2 infection. Vaccines are considered as the most effective approach for curbing infections [97,98,99,100] and are widely used for prophylaxis of several viral infections including influenza, measles, rubella, polio, etc. No suitable vaccine against SARS-CoV-2 has been approved so far. However, due to limited genetic variability and high resemblance with the SARS-CoV-1 genome, it is possible that a suitable vaccine against COVID-19 will be developed in a reasonable timeframe. For developing an effective vaccine, many researchers are currently trying to explore the structure of SARS-CoV-2 to find potential vaccine targets for this virus. SARS-CoV-2 is a single-stranded RNA virus (positive-sense); the 5′ end of its genome is comprised of an ORF1ab polyprotein, which encodes 15 or 16 non-structural proteins, while the 3′ end encodes four structural proteins including spike (S), membrane (M), envelop (E) and nucleocapsid (N) proteins, as depicted in Figure 2 [101,102].

### 7.1. Potential Vaccine Targets

Potential vaccine targets for SARS-CoV-2 include whole-cell antigens (WCAs) and structural proteins (S, M, E and N proteins). WCAs are comprised of all viral components including lipids, nucleic acids, proteins, polysaccharides, etc., and are used in the development of whole-cell killed/live-attenuated vaccines. A limitation associated with whole-cell antigens includes its complex composition, which causes issues in quality control and consistency assessment. For developing a WCA-based SARS-CoV-2 vaccine, strict screening of viral strains, with confirmed very low or no pathogenicity, is required [101].

The most potential antigen vaccine candidate for SARS-CoV-2 infection is S protein, because it includes surface exposure resulting in direct recognition by the host’s immune system. The structural S protein has been previously used for effective vaccine development against SARS/MERS CoVs and regulates interaction with the ACE2 receptor (responsible for virus entry and subsequent pathogenicity) of host cells. S protein is comprised of 2 subunits (S1 and S2). The S1 subunit depicts two domains, i.e., the C-terminal domain (CTD), which contains the RBD, and the N-terminal domain (NTD). Elements necessary for membrane fusion such as membrane proximal external region (MPER), trans-membrane domain (TM), internal membrane fusion peptide (FP) and 7-peptide repeats (HR) reside in the S2 subunit. The fragments of S protein including S1 subunit, FP, NTD, RBD and full-length S protein are promising candidates as antigens for vaccine development [103,104,105]. A DNA vaccine encoding full length S-protein of MERS CoV elicited a potent immune response in rhesus macaques, camels and mice [106]. Immunization of camels using adjuvanted S1 protein resulted in decreased and delayed MERS CoV shedding in the upper respiratory tract [107]. Recombinant RBD formulation in combination with aluminium (alum) adjuvant produced neutralizing antibodies in rhesus macaques, evident by the improvement of symptoms during MERS CoV infection [108]. Several antibodies against RBD are being investigated. By using existing anti-SARS drug candidates, for example, Tian et al. proved binding of the SARS-CoV-specific human monoclonal antibody (CR3022) with the SARS-CoV-2 RBD. No overlapping of the antibody epitope with the ACE2 linking site in the SARS-CoV-2 RBD represented that antibody neutralized the virus and inhibited its binding to the ACE2 receptor [109]. Lei et al. conducted an in-vitro study on antibodies against the ACE2 receptor of SARS-CoV-2. They produced a new recombinant protein having adequate affinity to the RBD of SARS-CoV-2 by linking the human ACE2 receptor to human immunoglobulin IgG1 (Fc region). This new protein effectively neutralized the SARS-CoV-2 virus [110]. Monoclonal antibodies offer specific drug targets compared to small-molecule drugs and therefore pose fewer side effects. Limitations associated with this therapy include the possibility that induced antibodies are involved in the pathogenesis of patients experiencing serious symptoms in addition to the neutralization of viral infection [51]. Tocilizumab, a monoclonal antibody, is also being investigated to treat SARS-CoV-2 infection [111,112,113]. One study proved the neutralizing activity of the NTD specified antibodies against MERS CoV, but as genomes of CoVs are variable, antibodies capable of targeting different epitopes should be used to avert virus escape from the immune system [114].

N protein, the most abundant protein found in CoVs, performs various functions such as RNA replication, mRNA transcription, formation of nucleocapsids and signal transduction virus budding. Reports have described this protein as having high antigenicity, as 89% SARS-CoV-infected individuals developed antibodies against this antigen, suggesting its potential use as a marker in diagnostic assays. M protein, abundant on the surface of SARS-CoV, plays a crucial role in virus assembly. Immunogenic and structural analysis indicated the presence of a T cell epitope cluster, on the transmembrane domain of this protein, capable of inducing a strong immune response. E protein, unlike M, S and N proteins, cannot be used as an immunogen due to its limited immunogenicity. Studies proved this protein of SARS-CoVs as a significant virulence factor. After knocking out E protein, reduction in the secretion of inflammatory factors (i.e., IL-6, TNF and IL-1 β) occurred [101,115].

### 7.2. Vaccines under Development

Currently, various types of SARS-CoV-2 vaccines including whole-cell killed, live attenuated, subunit, mRNA and DNA vaccines, live vector, synthetic peptide/epitope and virus-like proteins are under development. Whole cell killed/live attenuated vaccines offer numerous antigens to the host’s defence system, in turn, inducing a broad range of immunologic responses against the virus. The preparatory technology of this vaccine type has sufficiently matured with time [101,116].

Subunit vaccine involves one or more antigen potent enough to stimulate a host immune response. Advantages of this type of vaccine include its safety and easy production, but generally it requires mixing of an appropriate adjuvant to induce a strong immune response [101,117]. Any adjuvant already in use for other commercial vaccines can be reliably used for the SARS-CoV-2 vaccine, e.g., aluminium adjuvants are capable of eliciting a T-helper 2 (Th 2) cell immune response by increasing phagocytosis and decreasing antigen diffusion from the site of administration. Another adjuvant, MF59, generates a transient immune environment and recruits immune cells at the site of administration to stimulate an immune response. GlaxoSmithKline (GSK), Brentford, UK, developed a series of four adjuvants including AS01 (a liposome adjuvant), AS02, AS03 (an oil-in-water emulsifier) and AS04 (an aluminium adjuvant) [101]. Recently, GlaxoSmithKline (GSK) and Sanofi collaborated to develop an adjuvanted subunit vaccine. Sanofi provided its S-protein antigen (baculovirus production) based on recombinant DNA technology, while the fill and finish pandemic adjuvant system is being provided by GSK. Recently, the phase 1/2 clinical trial of this recombinant protein vaccine formulation was initiated [118,119].

No mRNA vaccine is commercially available in the market till date, but this type of vaccine can serve as a promising alternative to traditional vaccines due to its high potency, safe delivery, low-cost and short production cycle [120]. DNA vaccine, based on a plasmid DNA molecule encoding one or more antigens, is preferred over mRNA vaccines in formulations requiring administration efficiency and stability [121]. Contrary to most subunit vaccines, the ability of nucleic acid vaccines to elicit both the cellular and humoral immune responses make them promising vaccine candidates, although traditional attenuated vaccines are also capable of inducing both arms (cellular and humoral) of immune response; however, the possibility of reversion from the attenuated to the virulent state remains a limitation of this approach [122]. Delivery of nucleic acid vaccines by encapsulation in the polymeric or lipid matrices/nanoparticles and with the help of adjuvants has been successfully studied in order to circumvent different challenges such as the harsh digestive tract environment, enzymatic degradation and difficulty in crossing the phospholipid membrane (negatively charged) [123,124,125]. Chemical modification of RNA by pseudouridine can significantly enhance protein generation in-vivo by circumventing the protein translation inhibition, which is triggered by unmodified nucleotides [126]. Pardi et al. reported that nucleoside-modified mRNA vaccines can elicit potent T and B cell responses [127]. The potential of CpG-modified plasmid DNA vaccines for improving immunogenicity as compared to unmodified plasmid DNA is described in the literature [128]. The safety considerations regarding this vaccine type include induction of autoimmune responses and integration of vaccine into the host genome [129]. The regulatory concerns associated with nucleic acid vaccines are similar to those associated with all biological products, e.g., fabrication methods, design of plasmid constructs, source of DNA integrated into the vector including terminators, antibiotic resistance markers and promoters [130]. Jackson et al. conducted a phase 1 trial to study the efficiency of a candidate COVID-19 vaccine comprising of mRNA-1273 encoding SARS-CoV-2 S-protein. Participants in the study received two doses of the candidate vaccine (at day 1 and 28). The results showed that immune responses were elicited in all 45 participants without causing any trial-limiting adverse effects [131]. Another study was conducted by Corbett et al. to investigate an mRNA vaccine to non-human primates. The vaccine induced robust neutralizing activity and protection in airways with no pathological variations in the lungs [132]. Keech et al. delivered an NVX-CoV2373 nanoparticle vaccine, comprised of trimeric full-length SARS-CoV-2 S-protein with or without matrix-M1 adjuvant, to 131 healthy human subjects. By day 35, the prepared formulation appeared safe, and the adjuvanted vaccine showed ~100 times higher geometric mean fold IgG increases compared to that without adjuvant [133]. Currently, various mRNA vaccines are being developed to treat COVID-19 and would be delivered via the intramuscular route. The lipid nanoparticle (LNP)-based formulation approach is being adopted by various developers (~10) for delivery of mRNA vaccines, while intradermal or intramuscular delivery of DNA vaccines would be assisted by electroporation or needle-free injectors to improve gene transfection [134].

Live vector vaccines are comprised of live virus (vector) expressing heterologous antigens. This type of vaccine involves potent immunogenicity of live attenuated vector in combination with safety of subunit vaccines, capable enough to elicit a strong cellular immune response [101]. Human and chimpanzee adenoviruses are the two most commonly used vaccines vectors. The pre-existing immunity against human adenoviruses (specially serotype 5) results in decreased cellular and humoral immune responses against vaccine antigens. Several approaches, such as using alternative human serotypes, i.e., 26 or 35 serotypes, simian adenoviral vectors, chimpanzee adenoviral vectors and re-engineering the capsid of adenovirus 5 have been explored to avoid antibody recognition in order to overcome immune responses against vaccine antigens. Chimpanzee adenoviruses are safe and capable of stimulating both cellular and humoral immunity. However, vector-specific higher viral yields are desirable in order to obtain an optimal viral growth [135,136]. To overcome safety concerns regarding adenoviruses, viral genes including E1 (E1a and E1b), E2, E3 and E4 were removed, which resulted in replication deficient viruses and led to reduced cytotoxicity [137]. Alharbi et al. demonstrated the potential of S-protein-containing vector vaccines, based on chimpanzee adenovirus (ChAdOx1) and modified vaccinia virus ankara (MVA), against MERS CoV [138]. Zhu et al. assessed safety and immunogenicity of a non-replicating adenovirus type 5 vector COVID-19 vaccine candidate. This study demonstrated that the prepared vaccine was safe at a dose of 5 × 10¹⁰ viral particles/mL and elicited significant immune responses in the participants after immunisation [139]. Folegatti et al. administered adenovirus-vectored vaccine (ChAdOx1 nCoV-19) manifesting the SARS-CoV-2 S-protein to healthy adults, as a single dose, through the intramuscular route. Another group received two doses including a first and a booster dose. Spike-specific T-lymphocyte immune responses were at maximum on day 14. Anti-spike IgG responses were enhanced by day 28 and were boosted following a second dose. Neutralizing antibodies against SARS-CoV-2 were produced. Induction of cellular and humoral immunity, increased antibody response following the booster dose and a satisfactory safety profile suggested the potential of this candidate vaccine against SARS-CoV-2 infection [140]. Recently, both the ChAdOx1 nCoV-19 and adenovirus type 5 vector COVID-19 vaccine candidates have entered the phase 3 trial [134].

Synthetic peptide or epitope vaccines, comprised of fragments of intact antigens, are easy to formulate and evaluate, but their structural complexity as well as low molecular weight make them less immunogenic. This issue can be resolved by the addition of adjuvants or structural modifications [101]. Virus-like particles (VLPs) are promising vaccine candidates that resemble authentic viruses, in terms of molecular and morphological characteristics, but are non-infectious and non-replicating and hence can serve as promising vaccine candidates. Xu et al. developed SARS-CoV-2 VLPs by using the mammalian expression system. The structural proteins M and E played a major role in assembly and release of prepared VLPs, while S protein was highly incorporated on the VLP surface [141].

According to WHO (9 September 2020), currently >160 SARS-CoV-2 vaccine candidates are under development, in the clinical or pre-clinical evaluation stage, and a few examples are described in Table 3 [134].

## 8. Routes to Coronavirus Vaccine Delivery

An optimal route (in terms of practicability, safety and immunogenicity) is required for the appropriate administration of vaccines [151]. Various routes have been used for conventional vaccine delivery including the mucosal, parenteral and transcutaneous routes. This section deals with developments in the treatment of coronaviruses and therefore may offer potential in the future treatments of COVID-19.

### 8.1. Parenteral Immunization

Despite its invasive nature, conventionally the most widely used route for vaccine administration is parenteral (intramuscular, intradermal and subcutaneous). Coronavirus vaccines have been administered using subcutaneous and intramuscular routes. Tseng et al. administered four candidate SARS vaccines via the intramuscular route in a mouse model. The results showed induction of antibodies and protection from SARS infection by all vaccines, although hypersensitivity to vaccine components was observed [152]. Volz et al. administered a recombinant vaccine manifesting full length S glycoprotein of MERS CoV using intramuscular and subcutaneous injections in a mouse model. Virus-specific antibodies and CD8^+^ T lymphocytes were induced in mice, demonstrating efficacy of the administered vaccine [153,154]. Currently, several intramuscular and intradermal SARS-CoV-2 vaccines are being developed by various manufacturers [134].

### 8.2. Transcutaneous Immunization

The transcutaneous route is considered to be a more favourable mode for vaccine administration than intramuscular and subcutaneous routes, because skin layers are comprised of a relatively large number of antigen-presenting cells. In addition, it offers minimally invasive vaccine delivery and safer immune induction because there is no direct contact between potent/toxic adjuvants and the general circulation [155,156,157]. A major limitation associated with this route is skin layer stratum corneum; however, various approaches have been successfully explored to overcome this barrier [158,159]. Two approaches including electroporation and microneedles are promising candidates for transcutaneous administration of coronavirus vaccine. The electroporation technique has been commonly used for DNA vaccines [160,161]. Bernelin-Cottet et al. described the potential of the surface electroporation method for administration of plasmid DNA vaccine across pig skin. The vaccine stimulated recruitment of granulocytes, melanocytes and dendritic cells, in an association with local generation of IL-1β, IL-8 and IL-17 responses. Compared to microneedle patches and intradermal inoculation with a needle, robust and highest IFNγ T-cell and IgG responses were observed upon vaccine delivery via the surface electroporation method. The potential of electroporation-assisted intramuscular or intradermal administration of DNA vaccines for inducing strong immune responses has already been reported in literature [162]. Recently, microneedles (25–2000 µm) emerged as one of the most popular routes for vaccine administration due to several advantages, including self-application and cold chain independency (for proteins/vaccines/sensitive drugs) [163,164,165,166]. Three types of microneedle designs including coated, hollow and dissolving are being used for vaccine delivery [167,168]. Kim et al. prepared a dissolvable microneedle patch at room temperature with carboxymethylcellulose (CMC) using the spin-casting method and loaded recombinant coronavirus vaccine in needle tips. Microneedle-mediated SARS-CoV-2 S1 subunit vaccine delivery resulted in robust antigen-specific antibody responses, observed after 2 weeks of immunization, in a mouse model. Results proved that microneedles can serve as a potential candidate for the administration of recombinant protein subunit vaccines against coronavirus and other emerging infections [169].

### 8.3. Mucosal Immunization

Most of the infectious agents enter the human body through the mucosal route, as mucosal surfaces are thinner with high permeability. Vaccines administered by the mucosal route (intranasal/pulmonary, oral) can function more effectively by mimicking natural infection [170]. Limitations associated with this route include antigen instability at the mucosal site and difficulty in inducing an effective IgA antibody response (in practice) [171]. The oral route is the most compliant route for vaccine delivery purposes. Virus-like particles have been proven effective for oral vaccine delivery [170,172,173]. Various virus-like particle-based COVID-19 vaccines are under development [134]. Currently, an oral probiotic capsule-based SARS-CoV-2 vaccine is being established [174]. The nasal route is a potential route for immunization because it offers a large surface area with a thin mucous layer and high vascularisation [175]. In one study, mice were vaccinated intranasally with recombinant adeno-associated virus manifesting the RBD of SARS-CoV S-protein, and results were compared with intramuscular vaccination. The systemic humoral immune response generated by intranasal vaccine was comparable to intramuscular immunization in strength but had a shorter duration. Humoral response (local) and cytotoxic T lymphocyte responses (both local and systemic) were relatively stronger, and protection provided against SARS-CoV was comparable with intramuscular immunization. Increased mucosal IgA and neutralizing serum antibody titres were observed. The authors concluded that intranasal immunization may serve as a preferred route for SARS vaccine delivery due to its increased safety and ability to stimulate mucosal as well as systemic immunity [176]. Following pulmonary administration, the formulation approaches the respiratory tract, which offers an enlarged surface area, high permeability and abundance of antigen presenting cells including alveolar macrophages, dendritic cells and B cells. However, drug delivery to the alveolar regions of lungs is challenging, as the protective physiologic mechanisms and aerodynamics, coupled with particulate geometry, restricts the access of drug candidates to absorptive regions [170,177]. Currently, an inhaled vaccine formulation based on mRNA-encoded neutralising anti-COVID-19 antibodies, capable of producing antibodies directly in the lungs, is being developed [178].

## 9. Conclusions

Novel coronavirus infection has affected over 200 territories worldwide. Disease symptoms as well as the immune responses against this infection vary from individual to individual depending on several factors such as genetic traits, age and comorbidities. Several pre-existing antiviral agents have been repurposed for the treatment of COVID-19. However, this off-label use lacks reasonable evidence regarding efficacy of these agents. Some anti-inflammatory and antimicrobial agents are also being prescribed in order to manage co-morbidities/disease complications. Nevertheless, the role of vaccines to activate the immune system is of prime importance. Several strategies are in practice to develop vaccines including conventional (e.g., attenuated, whole cell killed) and advanced (e.g., LNPs based mRNA, DNA, VLPs and subunit vaccines). Clinical trials of multiple COVID-19 vaccine candidates, based on recombinant nanoparticles, mRNA and human and chimpanzee adenoviral vectors, have shown satisfactory results in early phases. It is expected that advanced technologies related to vaccine platforms, routes of administration, novel adjuvants, etc., will provide efficient, self-administrable and minimally invasive vaccine delivery leading to enhanced patient compliance and immunization coverage, especially in communicable diseases such as COVID-19 and in resource poor communities.

## Figures and Tables

**Table 1:** Symptoms of SARS-CoV-2 infection.

Patient Condition	Symptoms
Asymptomatic	RT-PCR test positive but no symptoms and chest imaging is normal
Mild	Digestive (diarrhoea, nausea/vomiting, abdominal discomfort) and acute upper respiratory tract infection (cough, fever, sore throat, fatigue, sneeze, myalgia and nasal congestion) symptoms
Moderate	Pneumonia without hypoxemia
Severe	Pneumonia with hypoxemia (SpO2 < 90% on room air) or respiratory rate >30 breaths/minute
Critical	ARDS, other symptoms include respiratory failure, cardiac injury, sepsis, septic shock, acute kidney injury and coagulation abnormality

**Table 3:** Examples of vaccines currently being developed for SARS-CoV-2.

Type of Vaccine	Platform	Current Stage and Trial ID	Manufacturer	References
Inactivated	Inactivated	Phase 3 (ChiCTR2000034780) Phase 1/2(ChiCTR2000032459)	Beijing Institute of Biological Products and Sinopharm	[134]
Inactivated	Inactivated	Phase 3 (NCT04456595) (669/UN6.KEP/EC/2020) Phase 1/2 (NCT04383574) (NCT04352608)	Sinovac	[134]
Whole-virion (inactivated)	Inactivated	Phase 1/2 (NCT04471519)	Bharat Biotech	[134]
Codon deoptimized live attenuated vaccine	Live attenuated virus	Pre-clinical	Indian Immunologicals and Griffith University	[142]
Microneedle patch (S1 subunit)	Protein subunit	Pre-clinical	University of Pittsburgh	[143]
Trimeric subunit (S protein)	Protein subunit	Phase 1 (NCT04405908)	Clover Biopharmaceuticals, GSK and Dynavax	[144]
Recombinant SARS-CoV-2 nanoparticle vaccine (full length glycoprotein) with/without matrix M as adjuvant	Protein subunit	Phase 2 b (NCT04533399) Phase 1/2 (NCT04368988)	Novavax	[145]
RBD protein fused with IgG (Fc region) in combination with an adjuvant	Protein subunit	Pre-clinical	Chulalongkorn University and GPO, Thailand	[134]
Molecular clamp stabilized S protein + MF59 adjuvant	Protein subunit	Phase 1 (ACTRN12620000674932p)	University of Queensland, CSL and Seqirus	[146]
Recombinant protein (RBD-dimer) with an adjuvant	Protein subunit	Phase 2 (NCT04466085) Phase 1(NCT04445194)	Anhui Zhifei Longcom Biopharmaceutical and Institute of Microbiology, Chinese Academy of Sciences	[134]
Recombinant protein, nanoparticles (containing S-protein and other epitopes)	Protein subunit	Pre-clinical	Saint-Petersburg scientific research institute of vaccines and serums	[134]
LNP-based peptide antigens	Protein subunit	Pre-clinical	IMV Inc	[134]
3 LNP-mRNAs	RNA	Phase 3 (NCT04368728) Phase 1/2 (2020-001038-36) (ChiCTR2000034825) (NCT04537949)	BioNTech, Pfizer and Fosun Pharma	[134]
LNP-encapsulated mRNA	RNA	Phase 3 (NCT04470427) Phase 2 (NCT04405076) Phase 1 (NCT04283461)	Moderna and NIAID	[147]
DNA plasmid vaccine (intradermal, followed by electroporation)	DNA	Phase 1/2 (NCT04447781) (NCT04336410)	Inovio Pharmaceuticals and International Vaccine Institute	[148]
Plasmid DNA (needle-free delivery)	DNA	Pre-clinical	Immunomic Therapeutics, EpiVax and PharmaJet	[149]
DNA plasmid vaccine with an adjuvant	DNA	Phase 1/2 (NCT04463472) (NCT04527081)	Osaka University, AnGes and Takara Bio	[134]
Para-influenza virus 5-based vaccine manifesting the S protein	Non-replicating viral vector	Pre-clinical	University of Georgia and University of Iowa	[150]
Intranasal recombinant vaccine based on Influenza A virus, for SARS-Cov-2 infection	Replicating viral vector	Pre-clinical	FBRI SRC VB VECTOR, Rospotrebnadzor and Koltsovo	[134]
Adenovirus type 5 vector	Non-replicating viral vector	Phase 3 (NCT04526990) (NCT04540419) Phase 2 (ChiCTR2000031781) Phase 1 (ChiCTR2000030906)	CanSino Biological and Beijing Institute of Biotechnology	[139]
ChAdOx1-S	Non-replicating viral vector	Phase 3 (ISRCTN89951424) (NCT04516746) Phase 2 (2020-001228-32) Phase 1/2 (PACTR202006922165132) (2020-001072-15)	University of Oxford and AstraZeneca	[140]
Enveloped virus-like particle	VLP	Pre-clinical	VBI Vaccines Inc.	[134]
